# Catalytic MOFs under Operation: From Static Descriptors to Dynamic Mechanistic Control

**DOI:** 10.1021/acs.accounts.6c00529

**Published:** 2026-09-24

**Authors:** Zheao Huang, Shaghayegh Naghdi, Pablo Ayala, Dominik Eder

**Affiliations:** † Institute of Materials Chemistry, 164718 Technische Universität Wien, 1060 Vienna, Austria

## Abstract

Metal–organic frameworks (MOFs) have traditionally been viewed as ideal heterogeneous catalysts because their crystallographic precision enables atomic-level structure–property correlations. Yet our work repeatedly showed that catalytic turnover rarely occurs on these idealized structures. Instead, active sites continuously evolve through substrate adsorption, transient coordination changes, charge redistribution, and surface reconstruction. The central challenge is therefore not simply designing stable frameworks but designing frameworks whose structural evolution remains mechanistically predictable. Therefore, how should structurally well-defined MOFs be interpreted as catalysts when their actual function depends not only on the as-synthesized lattice but also on this inevitable structural evolution during operation? We argue that this question extends beyond MOFs. Many modern heterogeneous catalysts—including oxides, single-atom catalysts, and coordination polymers—operate through dynamic active sites whose structure differs from the as-synthesized material. MOFs provide an unusually well-defined platform for understanding these transformations because both the starting structure and the subsequent evolution can be experimentally resolved. In this Account, we outline a necessary shift in MOF catalysis from treating static descriptors as universally predictive to controlling dynamic reaction mechanisms based on their elementary steps. To bridge this gap between idealized models and operational realities, we developed the framework-retaining selective ligand removal (SeLiRe) strategy. By exploiting the differential lability of mixed ligands, SeLiRe generates deterministic open metal sites and hierarchical pores across both hard–hard and soft–soft coordination networks without fully sacrificing the parent topology. We further investigated charge generation and extraction dynamics, demonstrating how modifications intended to improve optical absorption can inadvertently alter charge localization and introduce kinetic traps, while the dimensionality of inorganic secondary building units fundamentally dictates charge carrier lifetimes. Transitioning to harsher electrocatalytic regimes, we employ operando spectroscopy to capture the potential-driven evolution of these static open metal nodes. Specifically, we tracked the *in situ* transformation of low-valence sites into highly reactive, hydroxylated transient intermediate states alongside the controlled reconstruction of bulk frameworks into highly conductive surfaces. We propose that the principal advantage of MOFs is not that they remain structurally invariant during catalysis, but that they provide an experimentally accessible platform in which structural evolution can be designed, monitored, and mechanistically interpreted. This perspective shifts catalyst design from optimizing static descriptors toward engineering controlled dynamic active sites.

## Graphical abstract

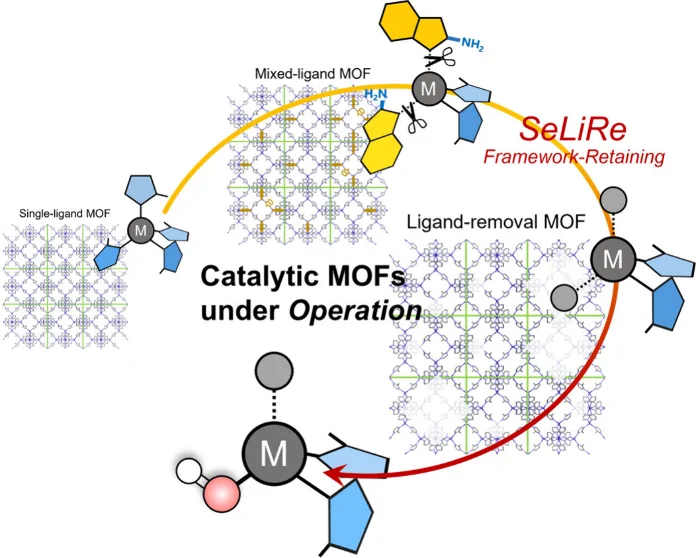

## Key References

-

Wang, J. ; Cherevan, A. S. ; Hannecart, C. ; Naghdi, S. ; Nandan, S. P. ; Gupta, T. ; Eder, D. Ti-based MOFs: New insights on the impact of ligand composition and hole scavengers on stability, charge separation and photocatalytic hydrogen evolution. Applied Catalysis B: Environmental 2021, 283, 119626 10.1016/j.apcatb.2020.119626 .^1^ *This study revealed that while amino-functionalization narrows the optical bandgap of Ti-MOFs, it inadvertently introduces deep hole-trapping states that severely suppress catalytic turnover*.
-

Naghdi, S. ; Cherevan, A. ; Giesriegl, A. ; Guillet-Nicolas, R. ; Biswas, S. ; Gupta, T. ; Wang, J. ; Haunold, T. ; Bayer, B. C. ; Rupprechter, G. ; Toroker, M. C. ; Kleitz, F. ; Eder, D. Selective ligand removal to improve accessibility of active sites in hierarchical MOFs for heterogeneous photocatalysis. Nat. Commun. 2022, 13­(1), 282 10.1038/s41467-021-27775-7 PMC875575235022390.^2^ *The targeted generation of hierarchical porosity and new active sites was achieved through a selective ligand removal strategy via thermolysis in mixed-ligand MIL-125. The controlled introduction of these distinct mesopore architectures drastically enhanced photocatalytic hydrogen evolution performance*.
-

Huang, Z. ; Wang, Z. ; Zhou, Q. ; Rabl, H. ; Naghdi, S. ; Yang, Z. ; Eder, D. Engineering of HO-Zn-N2 Active Sites in Zeolitic Imidazolate Frameworks for Enhanced (Photo)­Electrocatalytic Hydrogen Evolution. Angew. Chem., Int. Ed. 2025, 64­(7), e202419913 10.1002/anie.202419913 39688357.^3^ *The rational construction of coordinatively unsaturated open metal sites was achieved via a selective ligand removal strategy, wherein in situ tracking revealed a potential-driven dynamic evolution into high-valence HO–Zn–N*_*2*_ *moieties that effectively overcoming the limitations of water activation kinetic*.
-

Huang, Z. ; Wang, Z. ; Rabl, H. ; Naghdi, S. ; Zhou, Q. ; Schwarz, S. ; Apaydin, D. H. ; Yu, Y. ; Eder, D. Ligand engineering enhances (photo) electrocatalytic activity and stability of zeolitic imidazolate frameworks via in-situ surface reconstruction. Nat. Commun. 2024, 15­(1), 9393 10.1038/s41467-024-53385-0 PMC1152613039477934.^4^ *The operational durability and catalytic activity of ZIF-67 were synergistically enhanced through a ligand engineering strategy that directed a self-limiting surface reconstruction into a protective metal (oxy)­hydroxide layer while preserving the internal crystalline core*.

## 1 Introduction

Metal–organic frameworks (MOFs), assembled from metal nodes and organic linkers, occupy a distinctive position in heterogeneous catalysis because they combine precise structural definition with an exceptional degree of synthetic flexibility.^5,6^ Compared with conventional solid-state catalysts, MOFs offer clearer structural assignments and straightforward opportunities for atomic-level modification.^7–9^ Conversely, compared with isolated molecular systems, they provide robust, extended architectures with highly accessible internal surface areas. This inherent tunability naturally encouraged descriptor-based design strategies.^10–12^ In photocatalysis, linker functionalization is widely utilized to modify optical absorption, while pore architecture is tailored to enhance substrate accessibility.^13^ In electrocatalysis, conductivity, metal coordination environments, and bulk structural stability became similarly critical design criteria.^14–16^ However, this reliance on static structural descriptors introduces a core contradiction for MOF catalysis. Traditionally, MOFs are engineered for maximum rigidity and crystallographic perfection to ensure long-term stability. Yet, efficient catalysis is an inherently dynamic process that demands structural flexibility, such as transient bond breaking, local coordination changes, and active site evolution, to facilitate substrate turnover. While these static descriptors are useful for comparing related as-synthesized materials, they frequently fail to capture this dynamic reality of solid–liquid catalytic interfaces, where catalytic performance is largely governed by the continuous interplay between framework lability and operating conditions.^17–20^

Such interfacial dynamics introduce a fundamental tradeoff between structural robustness and catalytic function. In our own work, these issues emerged repeatedly across systems that at first appeared to raise unrelated questions, but ultimately pointed to a common theme: the catalytic behavior of MOFs must be interpreted in relation to the elementary processes that control turnover under realistic reaction conditions. For example, when investigating photocatalytic MOFs, we observed that structural modifications intended to improve one descriptor often inadvertently suppress overall catalytic turnover.^1,21^ While amino-functionalization improves visible-light absorption, it can also displace photogenerated holes away from the inorganic node, acting as deep charge traps that hinder interfacial reactions. Furthermore, efficient liquid-phase catalysis strictly demands the direct chemisorption of substrates onto catalytically relevant metal centers. In coordinatively saturated MOFs, this necessitates a dynamic opening of local metal–ligand bonds, rendering the framework highly susceptible to nucleophilic attack, solvent degradation, or metal leaching.^17,22,23^ Conversely, maximizing rigidity yields durable but inactive materials, whereas excessive lability leads to solvent degradation or metal leaching.^17,22,23^ While much of the field has shifted toward thermal carbonization or random defect engineering to circumvent these limitations, these nondeterministic strategies eradicate the pre-engineered local coordination that originally motivated the use of MOFs.^24–29^

As part of our ongoing research endeavors, our group has pursued “framework-retaining” ligand management strategies that preserve the topological determinism of the parent MOF while precisely manipulating its local coordination.^1–4,21,30–34^ The core of this methodology lies in the transition from mixed-ligand incorporation to selective ligand removal (SeLiRe). Inspired by Feng et al., who demonstrated the creation of hierarchical porosity in UiO-66 via controlled linker thermolysis,^35^ we exploit the differential labilities of distinct metal–ligand bonds to achieve the precise chemical cleavage of targeted coordination nodes across various MOF topologies. Crucially, our methodology enables the deterministic, atomic-level construction of coordinatively unsaturated open metal sites (OMSs) embedded within a rigid MOF matrix. Because interfaces are inherently dynamic, it is imperative to use *operando* characterization techniques to cross-interpret how the OMSs of these catalysts dynamically evolve under realistic reaction conditions.

This Account summarizes our efforts to bridge this gap, tracing MOF catalysis from macroscopic descriptors to atomic-level mechanisms (Figure 1). We first address the paradox between accessibility and stability in photoactive MOFs via the SeLiRe strategy. Next, we dissect charge transfer kinetics, revealing how ligand electronics and node dimensionality dictate carrier lifetimes rather than absolute optical absorption. Lastly, transitioning to harsh electrocatalytic conditions, we emphasize the imperative of operando characterizations to detail how static metal nodes dynamically evolve into high-valence active centers, and how rational ligand environments can deliberately guide *in situ* surface reconstructions. Ultimately, our objective is not to propose a dogmatic universal principle, but rather to highlight the mechanistic considerations we have found to be consistently imperative. We advocate for a shift in MOF catalysis from exploiting random defects and static descriptors toward the deterministic understanding and control of dynamic catalytic sites.

**Figure 1:**
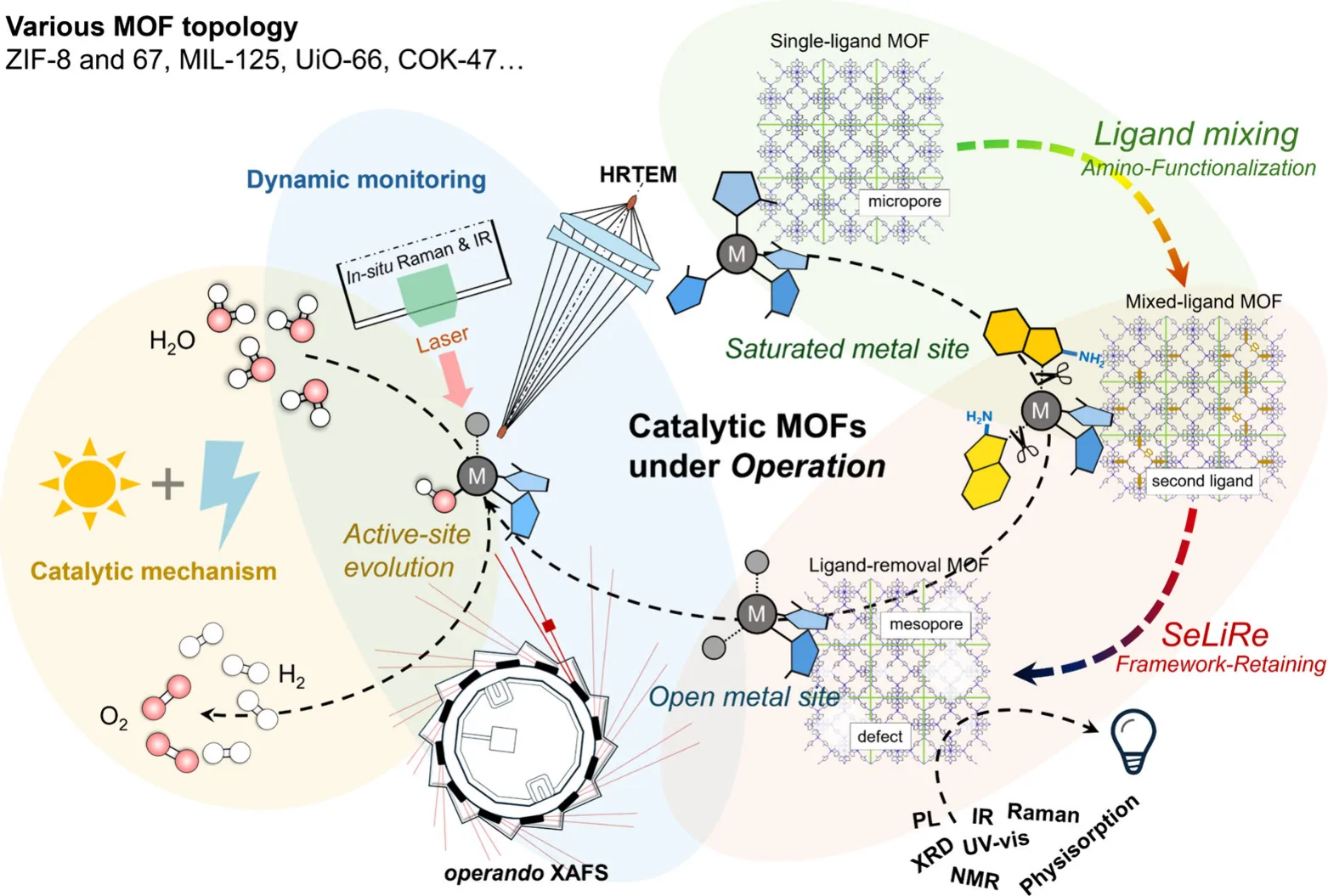
Schematic illustrating the evolution of catalytic MOF design from static to dynamic architectures. The progression shifts from conventional single-ligand frameworks to mixed-ligand systems, ultimately culminating in the SeLiRe strategy to engineer hierarchical mesopores and OMSs for explicitly tailoring structure–property–performance relationships in photo- and electrocatalysis.

## 2 Stability and Accessibility

MOFs are often attractive catalyst platforms precisely because their extended architectures and inorganic secondary building units (SBUs) are crystallographically well-defined.^36^ In practice, however, the requirements of catalysis in liquid media impose constraints that are not immediately apparent from structure alone. While optical absorption and applied voltage hints toward charge generation, the subsequent catalytic turnover is ultimately governed by charge separation, intra-SBU transport, and the chemical compatibility of the framework with the solvated reactants. Efficient turnover requires substrates to diffuse through solvated pore networks, access catalytically relevant SBU sites, adopt suitable adsorption geometries, undergo bond activation or charge transfer, and desorb without blocking further turnover.^37^ Concurrently, the MOF lattice must withstand dissolved electrolytes, applied bias, and sacrificial agents. This introduces a fundamental tension between structural robustness and catalytic function.

Titanium-based MOFs, such as MIL-125, attracted our attention because of their conceptual relationship with TiO_2_ (Figure 2a).^38–40^ At first glance, such systems seemed to offer an appealing combination of structural definition and photocatalytic relevance. Yet, the relationship between apparent stability and catalytic function proved less straightforward than expected.

**Figure 2:**
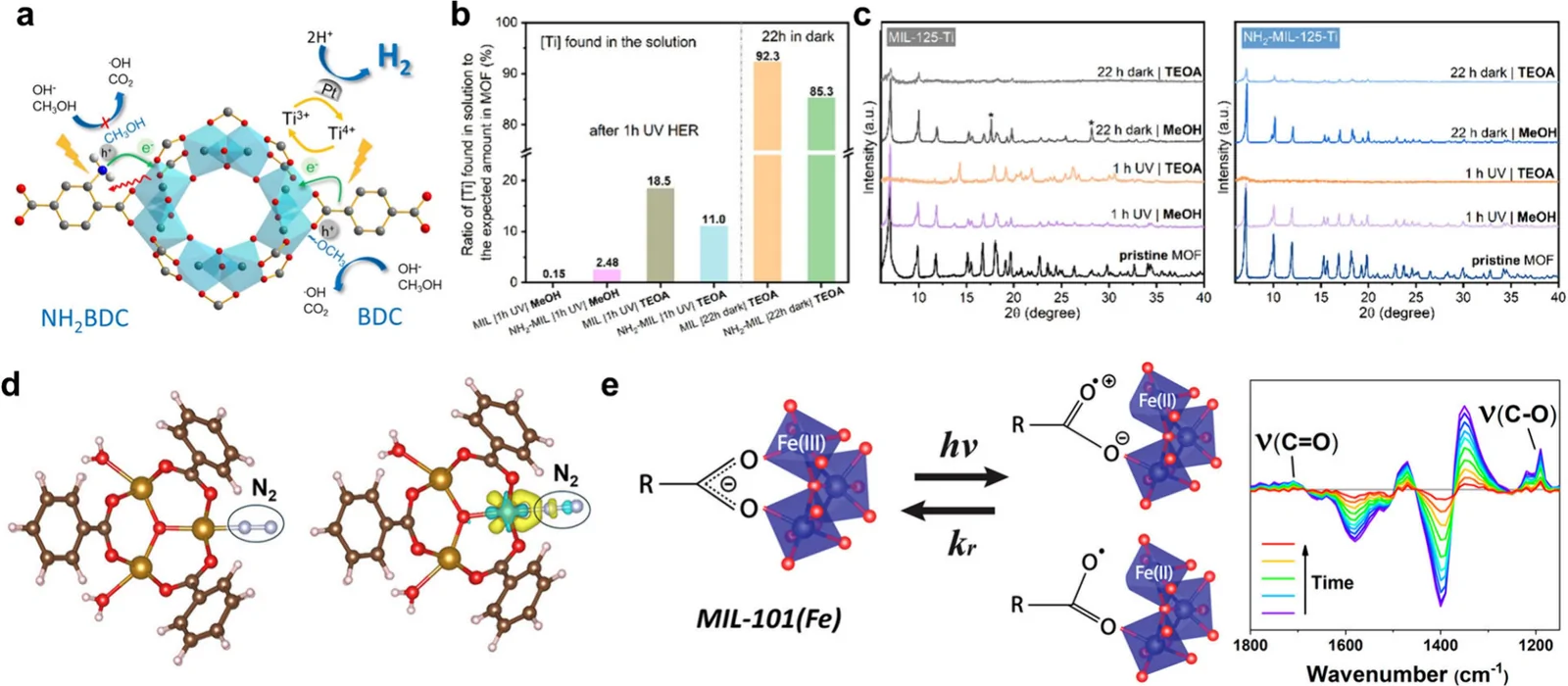
(a) Schematic illustration of charge generation, transfer, and recombination involved during photocatalytic hydrogen evolution in MIL-125-Ti-xNH_2_. (b) X-ray fluorescence (XRF) analysis quantifying titanium leaching into the reaction solution after 1 h of photocatalysis and after immersion in 0.01 M TEOA in the dark for 22 h. (c) Powder X-ray diffraction (XRD) patterns of the MOFs collected after photocatalysis utilizing different sacrificial donors (MeOH vs TEOA). Panels (a–c) reproduced with permission from ref 1. Copyright 2020 Elsevier B.V. (d) Density functional theory (DFT)-optimized model and charge density difference map of the Fe-MIL-101 secondary building unit, illustrating the end-on chemisorption of N_2_ at the transiently exposed Fe site. (cyan, charge depletion; yellow, charge accumulation). Reproduced with permission from ref 33. Copyright 2026 American Chemical Society. (e) Schematic representation of photoinduced dynamic ligation (PDL) in Fe-MIL-101 alongside time-resolved infrared (TRIR) difference absorption spectra collected at varied time delays. Reproduced with permission from ref 47. Copyright 2024 American Chemical Society.

When evaluating MIL-125 and its amino-functionalized analogue (NH_2_-MIL-125) for photocatalytic hydrogen evolution reaction (HER), stability depended strictly on the chemical environment.^1^ In the presence of triethanolamine (TEOA), a widely used sacrificial donor, significant degradation occurred. This was characterized by macroscopic framework corrosion and Ti^4+^ leaching, even under dark conditions (Figure 2b). Because TEOA acts as a competing ligand that displaces the native carboxylate linkers, leading to structural breakdown via ligand exchange, a process further accelerated by the alkaline pH (∼8) of TEOA solutions. This observation highlighted a critical mechanistic issue: sacrificial donors are often erroneously treated merely as operational additives, despite their direct and sometimes destructive influence on framework chemistry.^41,42^ Apparent stability cannot be extrapolated merely from *ex situ* structural assessments, nor can it be defined independently of the reaction environment. Rather, stability is an operational property determined by the elementary reaction mechanism and the chemical interactions required for substrate activation. Consequently, this realization necessitated a shift away from TEOA in favor of milder alcohol-based sacrificial agents like methanol (MeOH).^43–45^ Unlike TEOA, MeOH interacts only weakly with the organic linkers, merely adsorbing on the Ti-oxo clusters in a mildly acidic environment (pH ∼ 4.5) without disrupting the coordination bonds, under which both MOFs exhibited excellent structural retention (Figure 2c).

This divergence between TEOA and MeOH highlights a fundamental mechanistic principle in that many electro- and photocatalytic reactions proceed through direct charge (electron/hole) transfer to molecular reactants. This dictates that reactants must be chemically adsorbed directly on the metal site. With methanol as a sacrificial agent, MeOH forms methoxy groups directly on Ti sites, as evidenced by *in situ* infrared and X-ray photoelectron spectroscopies.^1^ Similarly, during solar ammonia synthesis over Fe-MIL-101, dinitrogen (N_2_) must directly adsorb onto the Fe centers (Figure 2d).^33^ In both cases, the active metal nodes are structurally coordinatively saturated in the ideal as-synthesized MOF. Therefore, for substrate adsorption to occur, the local coordination must partially open in a dynamic fashion, yielding transient open metal sites (OMSs). Recently, Morris et al. experimentally captured this phenomenon using time-resolved infrared spectroscopy, which they termed photoinduced dynamic ligation (PDL).^46,47^ Upon ligand-to-metal charge transfer (LMCT) excitation, the carboxylate-Fe bond in MIL-101 reversibly transitions from a symmetric bidentate bridging mode to an asymmetric monodentate configuration (Figure 2e). As demonstrated in our subsequent study, this transient deligation is precisely what provides the spatial and electronic access required for N_2_ to bind end-on at the temporarily exposed Fe centers.^33^

However, dynamic coordination opening is often not a degradation pathway, but an essential elementary step in catalysis. This introduces a fundamental stability-activity tradeoff: efficient catalytic turnover requires active-site accessibility, yet this accessibility frequently emerges from the same coordination dynamics that threaten structural stability. Macroscopic framework stability is often governed by the strength of these exact coordination bonds (guided by the hard–soft acid–base concept, alongside hydrogen bonding and π-π interactions).^48,49^ Consequently, the most structurally robust framework is not necessarily the most durable or effective catalyst under turnover conditions; rather, true catalytic function inherently requires controlled local lability. This tension between accessibility and coordination stability motivated our transition toward engineering accessibility more deliberately. Rather than relying on catalytic access emerging only through uncontrolled structural dynamics under reaction conditions, an alternative strategy is to design exceptionally stable MOFs and subsequently utilize postsynthetic processes to introduce accessible coordination environments in advance. This realization led directly to the development of our selective ligand removal strategy.

## 3 Selective Ligand Removal (SeLiRe)

Selective ligand removal was developed as a response to the limitations of accessibility generated through uncontrolled framework lability. Our solution to this paradox was to fundamentally separate catalyst synthesis from catalyst activation. If catalytic turnover depends on access to specific coordination environments, a robust design strategy must create such environments deliberately before catalysis begins, rather than relying on transient opening under reaction conditions. The SeLiRe strategy is therefore designed as a sequential two-step procedure: the strategic synthesis of a mixed-ligand MOF architecture followed by the targeted chemical cleavage of specific coordination bonds. To contextualize its utility, it is instructive to briefly compare SeLiRe with other prevalent defect engineering strategies.^29,50,51^ Presynthetic modulated synthesis introduces missing-linker defects during crystallization, yet these are often stochastically distributed. Etching approach efficiently creates hierarchical porosity but frequently causes irreversible collapse of local coordination geometries. Solvent-assisted linker exchange can achieve compositional control but is primarily designed for ligand substitution rather than generating targeted vacancies. In contrast, SeLiRe provides a deterministic, framework-retaining route. Driven by coordination chemistry, our objective is the chemically directed modification of these nodes to create persistent, rather than transient and degradative, active site accessibility.

The central requirement for SeLiRe is the coexistence of ligands with sufficiently distinct chemical stability to create a window in which one linker can be removed preferentially while the larger framework remains intact. Whether such a window exists depends strongly on the underlying coordination chemistry. In carboxylate-based MOFs, this condition is achieved comparatively readily because framework stability is dominated by hard–hard acid–base interactions. Titanium and zirconium centers strongly favor oxygen donor ligands, providing substantial thermodynamic stabilization. Early investigations into framework engineering through the partial ligand removal were pioneered in the Zr-UiO-66 system, illustrating the potential of missing-linker defects to modulate internal porosity (Figure 3a).^35,52,53^ A few years ago, we initially validated and expanded this concept within the Ti-MIL-125. By synthetically varying the incorporation ratios of standard terephthalic acid (1,4-benzenedicarboxylate, BDC) against the more thermolabile amino-terephthalic acid (2-amino-1,4-benzenedicarboxylate, BDC-NH_2_), we assembled a series of mixed-ligand (ML) MIL-125 frameworks.^2^ Through *in situ* diffuse reflectance infrared Fourier transform spectroscopy (DRIFTS) and thermogravimetric analysis, we identified a critical thermal window (∼300 °C) where the targeted metal–ligand coordination bonds of BDC-NH_2_ were selectively cleaved while the BDC linkages remained intact (Figure 3b).

**Figure 3:**
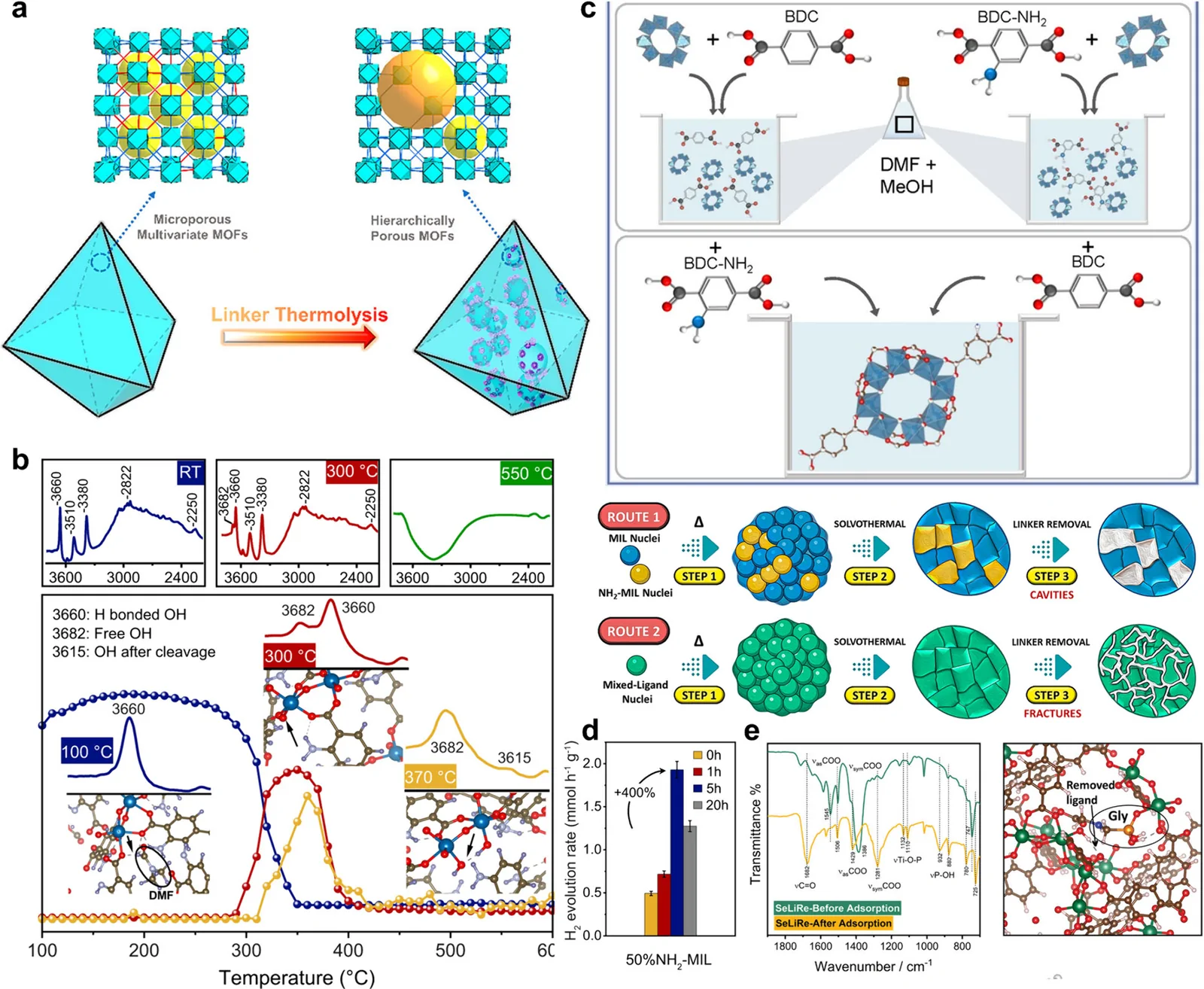
(a) Schematic diagram illustrating the transformation of microporous UiO-66-NH_2_ into hierarchically porous MOF through linker thermolysis. Reproduced with permission from ref 35. Copyright 2018 American Chemical Society. (b) Selected *in situ* DRIFTS bands and their corresponding thermal evolution plots, identifying the critical processing window required for the selective cleavage of thermolabile linkers. (c) Schematic representation of the synthetic routes for mixed-ligand MIL-125 utilizing different ligand addition sequences, which deliberately preprogram the spatial distribution of the removable domains. (d) Photocatalytic H_2_ evolution rates or pre- and post-thermolysis MOFs. Panels (b–d) reproduced with permission from ref 2. Copyright 2022 Springer Nature. (e) Infrared spectroscopic studies of the SeLiRe-processed MOFs following glyphosate adsorption. The emergence of distinct vibrational bands (932, 880, 1132, and 1110 cm^–1^) verifies the targeted monodentate coordination of glyphosate at the newly generated Ti open metal sites, accompanied by a schematic illustration of this specific adsorption geometry. Reproduced with permission from ref 30. Copyright 2023 Wiley-VCH GmbH.

This process highlighted a broader issue that extends beyond selective ligand removal itself: *average framework composition is often an incomplete structural descriptor*. In mixed-ligand MOFs, nominal stoichiometry does not reveal whether linkers are statistically dispersed or locally clustered, yet these local distributions dictate the ultimate outcome of postsynthetic transformations. For example, by modulating the sequence of ligand introduction, we preprogrammed the spatial distribution of these domains (Figure 3c). This synthetic control is fundamentally governed by differential binding affinities of the ligands. Because BDC exhibits a stronger coordination affinity to the titanium nodes than BDC-NH_2_, the order of ligand addition dictates the framework assembly pathway. When BDC is added first, it forms 5–10 nm nuclei, while subsequent addition of BDC-NH_2_ generates separate nuclei; their agglomeration produces particles containing distinct BDC-NH_2_-rich domains that, upon thermal removal, evolve into isolated cavities. When utilizing a reverse sequence, the stronger BDC triggers a partial ligand exchange during nucleation, resulting in a homogeneous dispersion of the thermolabile linkers across the framework. Upon thermal treatment, removing these homogeneously dispersed linkers generated interconnected fracture-type pathways. These fractures minimized intracrystalline diffusion distances, leading to a 400% enhancement in photocatalytic HER rates (Figure 3d).

Conversely, adding the thermolabile BDC-NH_2_ linker to a premixed BDC solution induces localized clustering. Subsequent thermolysis of these clustered domains generated isolated cavity-type pores. These cavity-rich architectures proved highly effective for the specific adsorption of large molecules like glyphosate.^30^ Mechanistic analysis revealed that the engineered cavities allowed glyphosate to coordinate with the newly exposed Ti open metal sites through a distinct monodentate configuration (Figure 3e), permitting fully reversible adsorption–desorption cycling. This confirms that SeLiRe extends beyond mere mass transport engineering; it enables deliberate, substrate-specific active-site design.

## 4 Open Metal Sites and Isolated Active Centers

The SeLiRe therefore changes the role of the MOF. Rather than serving as a precursor for pyrolysis, the framework becomes an atomically programmable scaffold that defines both active-site geometry and mass transport. To demonstrate the universality of our approach, we sought to extend the SeLiRe concept from the highly ionic “hard–hard” coordination networks of carboxylate MOFs to the softer coordination environments of zeolitic imidazolate frameworks (ZIFs; Figure 4a).^54,55^ In the ZIFs, the “soft–soft” Metal–N linkages possess greater covalent character, generating a critically narrow thermolysis temperature window (Figure 4b).

**Figure 4:**
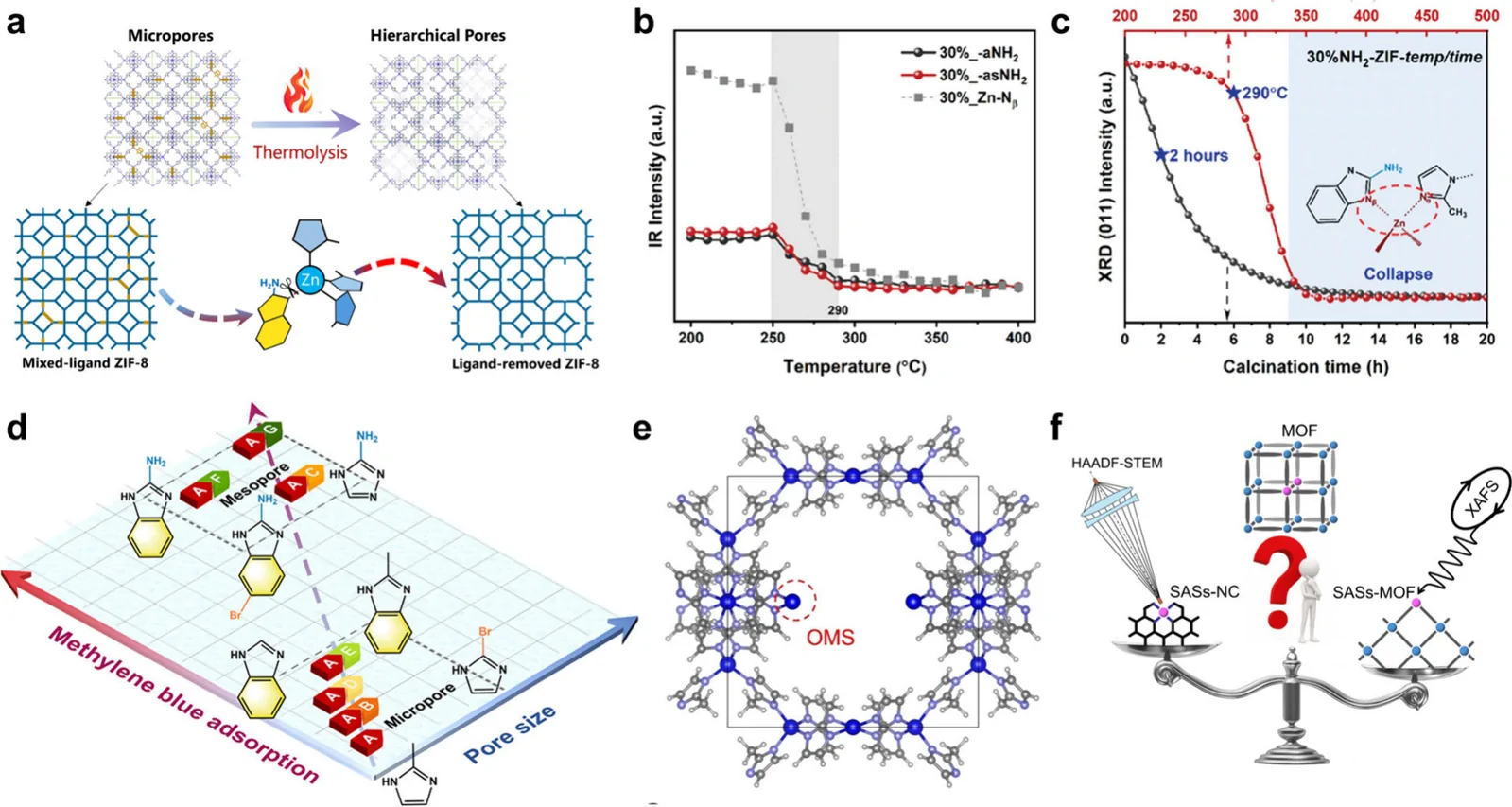
(a) Schematic diagram of the SeLiRe process within the ZIF framework. (b) Infrared spectroscopic evolution of the ν­(−NH_2_) and ν­(Zn–N_β_) bands for mixed-ligand ZIFs upon heating. (c) *In situ* X-ray diffraction (XRD) evolution intensity plot of the (011) peak as a function of calcination temperature and duration. Panels (a–c) were reproduced with permission from ref 31. Copyright 2023 Wiley-VCH GmbH. (d) Schematic diagram illustrating the relationship among secondary ligand selection, pore-type construction, and enhanced water purification capacity (methylene blue adsorption). Reproduced with permission from ref 32. Copyright 2026 American Chemical Society. (e) Optimized model structures of the open metal site ZIF (OMS-ZIF) following targeted ligand removal, showing the intentionally low-valence Zn–N_2_ configuration. Reproduced with permission from ref 3. Copyright 2025 Wiley-VCH GmbH. (f) Schematic diagram comparing conventional pyrolyzed M–N–C single-atom sites (SASs-NC) with framework-retained single-atom sites (SASs-MOF). Reproduced with permission from ref 28. Copyright 2026 American Chemical Society.

By utilizing mixed-ligand (ML)-ZIF-8 comprising 2-methylimidazole (2-mIm) and the thermolabile 2-aminobenzimidazole (NH_2_-bIm), we successfully executed SeLiRe without triggering the collapse of the inherent sodalite topology.^31^ By spectroscopically monitoring the NH_2_ groups and Zn–N stretching vibrations, we confirmed the selective and quantitative cleavage of Zn–N bonds associated with NH_2_-bIm. *In situ* temperature-dependent XRD verified the preservation of the crystalline sodalite framework during this delicate thermal cleavage (Figure 4c), with forming the uniform mesopores (2–30 nm). This structural opening increased the adsorption capacity for bulky molecules like methylene blue by forty-fold, an effect that holds true across various imidazole ligands (Figure 4d).^31,32^

More importantly for catalysis, it intentionally reduced the saturated tetrahedral Zn–N_4_ geometry to an exposed, low-valence Zn–N_2_ configuration (Figure 4e). The catalytic relevance of these open metal sites depends strongly on the surrounding framework chemistry. In this system, coordinative unsaturation does not simply improve physical access to an otherwise buried active site; it fundamentally changes the chemistry of the material. Lowering the coordination number directly alters the local electronic structure of the zinc center, facilitating interaction with substrates such as hydroxide ions, and substantially lowering the energetic barrier for water activation under reaction conditions. In this sense, selective ligand removal generated not merely a more accessible catalyst, but a chemically distinct active-site environment.

This transition from accessibility engineering to active-site engineering provides a conceptual connection to single-atom catalysis. Many state-of-the-art single-atom catalysts rely on carbon-supported M–N–C motifs generated through high-temperature pyrolysis. While these systems often achieve impressive catalytic performance, structural interpretation is rarely straightforward due to heterogeneous active-site distributions and uncontrollable support evolution under operation.^28,56^ Framework-retained isolated sites represent a fundamentally different design space, which itself remains structurally relevant to catalysis (Figure 4f). By sufficiently constraining the local chemistry, this approach overcomes the heterogeneity of pyrolyzed catalysts, preserving strict site isolation while enabling high catalytic activity. Ultimately, retaining this precise control over local coordination geometry and pore accessibility allows us to explicitly relate atomic-level structural features to macroscopic catalytic function.

However, realizing this broader design principle, i.e. programming postsynthetic transformations rather than introducing them statistically, requires recognizing that SeLiRe is not universally applicable. Defining its limitations is therefore essential. Purely thermal SeLiRe is expected to fail for frameworks with intrinsically low stability, highly oxophilic high-valent or rare-earth metals, or metal–ligand bonds of comparable lability. For example, whereas Zn–N bonds in ML-ZIF-8 provide a viable thermal cleavage window, applying the same protocol to the isostructural ZIF-67 leads to framework collapse because the unsaturated Co–N bonds lack sufficient thermal stability.^31^ Of course, SeLiRe is not fundamentally restricted to thermolysis. When a thermal window is prohibitively narrow, this limitation can be circumvented through orthogonal activation routes. Future optimizations to broaden the applicability of SeLiRe include incorporating photolabile linkers cleavable via targeted irradiation or utilizing solvent-mediated chemical excision. We anticipate similar strategies could ultimately be extended to covalent organic frameworks and hybrid coordination polymers.

## 5 Charge Carrier Dynamics

Photocatalytic MOFs are often discussed in terms of optical descriptors such as apparent band gaps, and surface area/porosity maximization.^57–60^ While these quantities are useful when comparing related materials, their catalytic significance ultimately depends on whether the generated charge carriers can participate productively in the relevant chemistry. Our work repeatedly highlighted the distinction between light absorption, charge separation, and charge extraction.

Fundamentally, a narrowed band gap does not inherently equate to catalytic activity; rather, charge localization profoundly outweighs absolute optical absorption. For instance, in photocatalytic HER, pristine MIL-125 outperformed its amino-functionalized analogue (NH_2_-MIL-125) by a factor of ∼ 40, despite the latter’s superior visible-light absorption (Figure 5a).^1^ Transient spectroscopy and density functional theory revealed why: while photoexcitation in both MOFs generate Ti^3+^ centers via ligand-to-metal charge transfer, the spatial distribution of the holes diverges (Figure 5b). In pristine MIL-125, holes are located near the Ti-oxo clusters, enabling rapid methanol oxidation. Because methanol oxidation proceeds through adsorbed methoxy-type intermediates directly at these titanium sites, this close proximity enables rapid charge extraction. Conversely, NH_2_-MIL-125 deeply traps holes at the linker’s nitrogen atoms, creating a severe kinetic barrier that negates the benefits of improved absorption.

**Figure 5:**
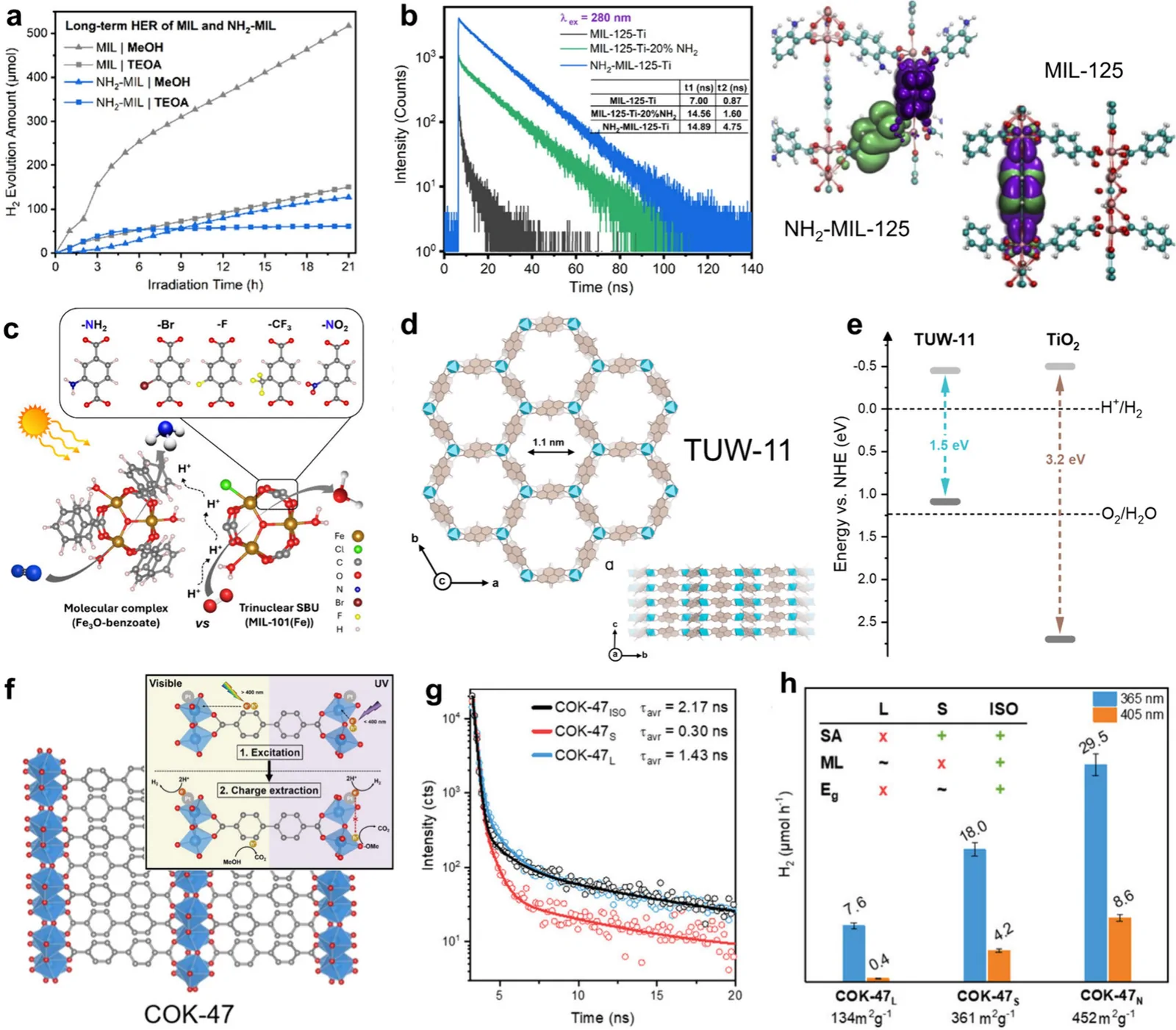
(a) Photocatalytic H_2_ generation upon UV irradiation of MIL-125-Ti-xNH_2_ with different hole scavengers. (b) Time-resolved emission spectra in aqueous MeOH alongside isodensity representations of the photoexcited interacting charge carriers (electrons in violet, holes in green). Panels (a) and (b) are reproduced with permission from ref 1. Copyright 2020 Elsevier B.V. (c) Schematic representation of ligand functionalization utilized to tune the electronic and steric environment of the Fe-MIL-101 SBU. Reproduced with permission from ref 33. Copyright 2026 American Chemical Society. (d) Structural models of TUW-11 (Technische Universität Wien) displaying the layered architecture assembled through π–π interactions along the *a* axis and the microporous 1.1 nm 1D channels along the *c* axis. (e) Energy diagram showing the HOCO/LUCO alignment of TUW-11 relative to TiO_2_. Panels (d) and (e) are reproduced from ref 62. (f) Schematic illustration of the extended 2D COK-47 structure, where continuous inorganic sheets enable band-like electronic transport. (g) Time-resolved photoluminescence (PL) emission spectra of COK-47 materials under 377 nm excitation. (h) H_2_ evolution rates demonstrate the severe catalytic penalty induced by missing-ligand (ML) defects. Panels (f–h) reproduced with permission from ref 21. Copyright 2023 Wiley-VCH GmbH.

Importantly, this effect is mechanistically context-dependent. When sacrificial donors like triethanolamine are used, radical-mediated pathways dominate, rendering the exact location of the photogenerated hole less critical, which explains why amino-functionalized MOFs often excel in other photocatalytic systems.^13,61^ However, when direct interfacial charge transfer is rate-limiting, charge localization becomes a severe penalty. A similar phenomenon occurred in our Fe-MIL-101 system for N_2_ reduction, where amino groups trapped holes and suppressed local proton availability—a bottleneck we resolved via strategic fluorination (Figure 5c), which prolonged excited-state lifetimes and boosted NH_3_ production by ∼ 60%.^33^

Beyond initial charge generation and localization, the dimensionality and connectivity of the inorganic SBU strictly dictates transport and recombination. For example, TUW-11 features isolated 0D Ti^4+^ nodes within a highly conjugated ligand network (Figure 5d; a titanium MOF from our group synthesized from sustainable plant-based ellagic acid).^62^ Despite an ideal bandgap (Figure 5e), its lack of direct Ti–O–Ti connectivity prevents Ti^3+^ generation, leading to rapid recombination and negligible HER activity.

While 0D octameric clusters (e.g., MIL-125) permit local electron delocalization, macroscopic charge extraction requires continuous 2D inorganic sheets, as realized in COK-47.^63^ Comprising infinite 2D TiO_6_ layers (Figure 5f), COK-47 enables band-like electronic transport. However, this extended connectivity is highly sensitive to defects. In isotropic COK-47 nanoparticles, random missing-ligand defects acted as deep recombination centers, slashing carrier lifetimes from 2.17 to 0.30 ns (Figure 5g) and decreasing activity 7-fold (Figure 5h).^21^ Thus, the transport benefits of 2D connectivity are instantly lost if structural defects trap migrating charges.

Collectively, these studies suggest that photocatalytic descriptors must be ranked hierarchically. Optical absorption determines whether carriers are generated, but catalytic performance ultimately depends on where those carriers localize and whether they can reach reactive interfaces before recombination. This hierarchy provides a far more predictive framework than traditional band gap engineering.

## 6 Structural Evolution under Operation

Electrocatalytic systems introduce an additional complication: *the catalytically relevant structure often emerges only under operating conditions*. Applied potential, localized pH gradients, and sustained current flow force the framework to undergo adaptive structural evolution.^28^ These are predominantly driven by the adsorption or direct coordination of oxygenated intermediates (e.g., *O, *OH, and *OOH), and excessive electro-oxidation.^64–67^ Consequently, MOFs must routinely be classified as precatalysts in these regimes.^17,22^ Structurally well-defined precursors do not eliminate the need to identify these catalytically relevant states; rather, catalytic interpretation requires understanding both the initial architecture and the evolution pathway that occurs during operation. While catalyst reconstruction has become widely recognized, much less attention has been devoted to controlling reconstruction pathways.^68^ We argue that the relevant design objective is therefore not preventing structural evolution but directing it toward chemically interpretable active states.

This distinction is particularly important for systems containing isolated metal sites. Using our SeLiRe-engineered OMS-ZIF under alkaline electrocatalytic HER conditions,^3^ the *ex situ* characterization established the presence of low-coordinate zinc environments, but operando electrochemical Raman spectroscopy showed the emergence of distinct HO–Zn vibrational signals. Concurrently, highly coordinated sensitive X-ray absorption spectroscopy supported the fitting of a new high-coordination Zn–O scattering path (Figure 6a). These real-time observations verified that the static Zn–N_2_ nodes underwent a potential-driven transformation into transient, high-valence HO–Zn–N_2_ active moieties (Figure 6b). Because static calculations on idealized structures rarely capture dynamic environments relevant to electrocatalysis, density functional theory calculations explicitly incorporating these adsorbates corroborated that this *in situ* hydroxyl chemisorption reconfigures the localized electronic structure of the zinc center. Stabilized by the rigid framework, these hydroxylated sites lowered the energy barrier for water activation, achieving a current density of 1.0 A cm^–2^ over 120 h of operation.

**Figure 6:**
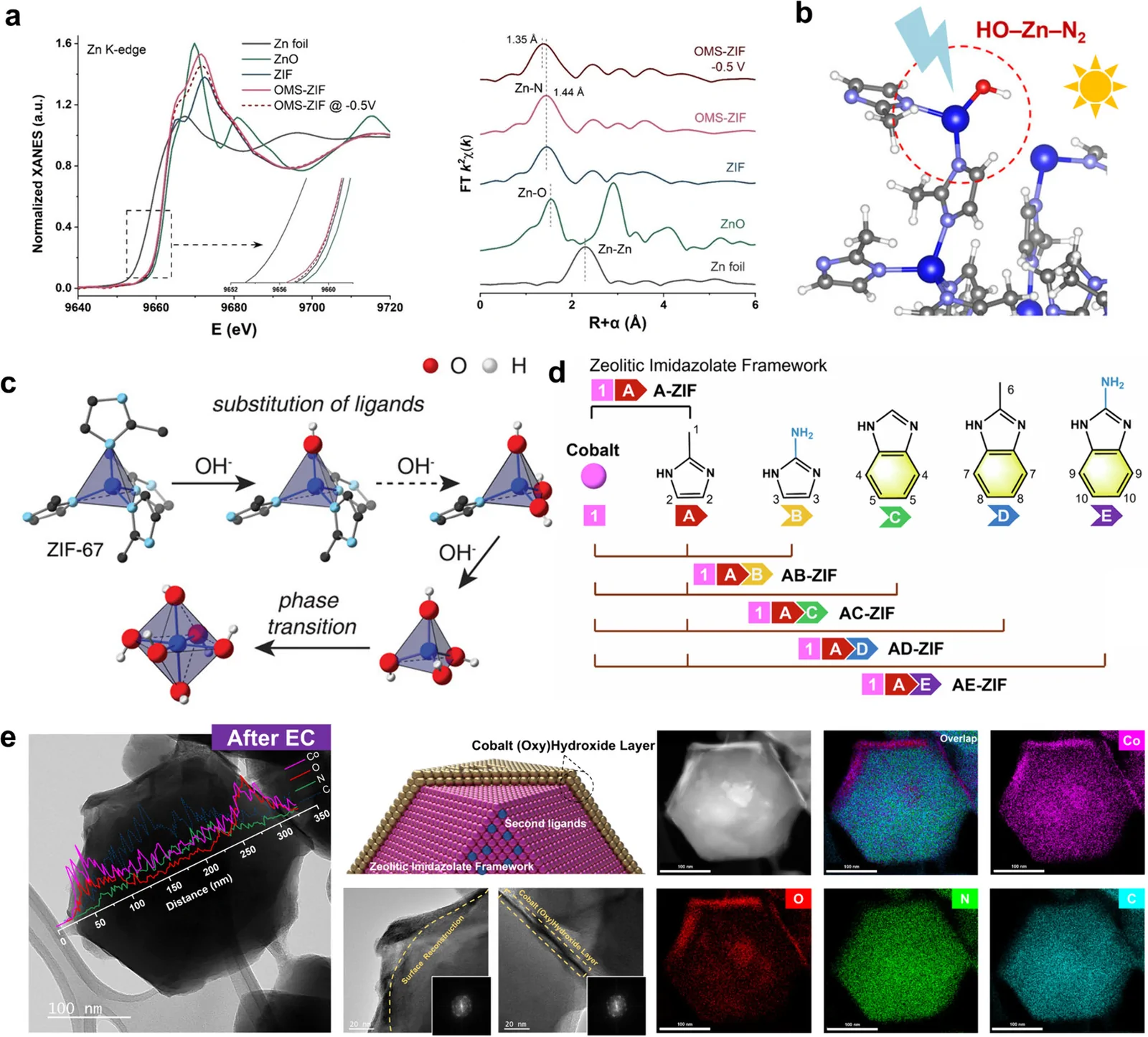
(a) Zn K-edge X-ray absorption near-edge structure (XANES) spectra and Fourier-transformed (FT)-extended X-ray absorption fine structure (EXAFS) analysis of OMSs in ZIF samples. (b) Optimized model of the HO–Zn–N_2_ active sites in the OMS-ZIF. Panels (a) and (b) are reproduced with permission from ref 3. Copyright 2025 Wiley-VCH GmbH. (c) Illustration of the uncontrolled precatalytic conversion of pristine ZIF-67 into bulk α/β-Co­(OH)_2_. Reproduced from with permission ref 22. Copyright 2019 American Chemical Society. (d) Schematic diagram of multivariate ligand incorporation in ZIF-67. (e) Transmission electron microscopy (TEM) images with a corresponding line scan and elemental mapping of a mixed-ligand ZIF-67 particle following OER amperometry. Panels (d) and (e) reproduced with permission from ref 4. Copyright 2024 Springer Nature.

Under harsher anodic regimes, such as the oxygen evolution reaction (OER), potential-driven transformations frequently encompass the bulk framework. For example, Zheng et al. demonstrated that pristine cobalt-based ZIF-67 experiences an irreversible conversion into amorphous or poorly crystalline cobalt hydroxides (α/β-Co­(OH)_2_) phases during OER (Figure 6c).^22^ These phases subsequently serve as the dominant active sites, rather than the intact MOF nodes. This identical overoxidation pathway affects numerous transition metal-based MOFs (e.g., Co, Ni, Mn, and Fe), a vulnerability previously articulated by Zhang et al.^23^ and corroborated by our group’s research.^4^ In the broader field, structural persistence is sometimes treated as the defining criterion for catalyst quality, while reconstruction is sometimes interpreted uncritically as activation. However, structural persistence and complete reconstruction should not be viewed as opposing design philosophies. Instead, both represent limiting cases within a broader continuum of catalyst evolution. As articulated in our recent perspective article,^17^ the key question becomes whether the evolving structure remains chemically constrained and mechanistically interpretable.

By employing multivariate ligand incorporation in ZIF-67, we proactively guided this macroscopic reconstruction.^4^ The local chemical environment, modulated by the supplementary amino groups and benzene rings of the mixed-ligand system (Figure 6d), restricted the structural reconstruction to the outermost surface. This yielded a highly conductive metal (oxy)­hydroxide layer that encapsulated the intact parent MOF architecture (Figure 6e). This core–shell configuration fulfills a dual purpose by providing a synergistic interfacial effect to promote electron transfer, while acting as a physical barrier that prevents the electrolyte from further attacking the internal crystalline core. Comparable stabilization strategies apply to nickel- and iron-based MOFs; for instance, integrating the elongated BPDC (4,4′-biphenyldicarboxylate) linker mitigates bulk self-reconstruction, promoting the controlled formation of surface metal hydroxide phases.^69–71^ The relevant design criterion is therefore mechanistic control, rather than structural immutability.

## 7 Conclusions and Outlook

Over the past decade, our investigations across diverse photocatalytic and electrocatalytic systems, spanning Ti, Fe, Zn, and Co based frameworks, have repeatedly led to the same conclusion that catalytic performance cannot be understood solely from the static structure of the as-synthesized material. While these investigations initially appeared disconnected, a consistent set of mechanistic principles emerged.

A first recurring observation is that static descriptors are often necessary but insufficient predictors of catalytic activity. Optical bandgap, porosity, coordination geometry, and apparent structural stability remain valuable parameters for comparing related materials. However, these descriptors frequently fail to predict catalytic turnover because they do not directly capture the processes that govern charge extraction, substrate activation, or active site evolution. Static descriptors therefore explain catalyst structure, whereas catalytic performance ultimately depends on dynamic descriptors associated with charge localization, adsorption, transport, and structural evolution (Table 1).

**Table 1:** Summary of Representative MOF/ZIF Systems Comparing Expected Outcomes Based on Tailored Static Descriptors with Actual Performances^a^

MOF System (Comparison)	Tailored Static Descriptor	Expected Outcome	Actual Performance	Governing Dynamic Factor
Ti-MIL-125 vs NH_2_-MIL-125 ^1^	Narrowed Eg (2.8 vs 3.8 eV); high S_BET_	Higher HER for NH_2_-MIL due to visible-light absorption	MIL-125 HER rate ∼40× higher (58.4 vs 1.5 μmol h^–1^)	**Charge trapping**: Holes trapped at N atoms block MeOH scavenging; MIL-125 permits direct transfer (proven by PL/TRES).
Pristine vs SeLiRe MIL-125 ^2,30^	40–50% drop in S_BET_ and microporosity	Lower HER/Ads. due to reduced static surface area	∼5× HER increase; ∼3× Gly Ads. capacity increase	**Accessibility**: 3D mesopores eliminate diffusion limits; newly exposed Ti–OH sites override low S_BET_.
Pristine vs Defective COK-47 ^21^	Missing ligand defects (0% in pristine COK-47_ISO_ vs 32% in defective COK-47_S_)	Defects increase porosity and facilitate access to active sites, boosting HER	Defect-free COK-47_ISO_ shows ∼2× higher vis-HER than defective COK-47_S_ (8.6 vs 4.2 μmol h^–1^)	**Charge Recombination:** Defects act as kinetic traps rather than new pores; carrier lifetime slashed from 2.17 to 0.30 ns.
SL-ZIF vs LE-ZIF *(Surface Reconstruction)* ^4^	Ligand composition (single 2-mIm vs mixed-ligands with aromatic/amino groups)	Complete reconstruction into CoOOH and loss of framework/porosity during OER	LE-ZIF exhibits 10× higher OER current and extreme stability over 12 h compared to SL-ZIF	***In-situ*** **Surface Reconstruction**: Formation of a protective CoOOH outer layer preserves the inner MOF core and prevents CR.
Saturated ZIF vs OMS-ZIF *(Site Evolution)* ^3^	Metal coordination (Saturated Zn–N_4_ vs unsaturated Zn–N_2_ via SeLiRe)	Saturated coordination restricts catalytic kinetics in alkaline media	OMS-ZIF needs 0.07 V for 10 mA cm^–2^ vs 0.31 V for ZIF-8; achieves ampere-level HER (1.0 A cm^–2^) at 0.41 V	**Operando Site Evolution**: Zn–N_2_ chemisorbs OH^–^ to form high-valence HO–Zn–N_2_ sites, lowering H_2_O dissociation energy (Volmer step).

a **Abbreviations**: *S*_BET_: Brunauer–Emmett–Teller specific surface area; SL-ZIF/LE-ZIF: single-ligand/ligand-engineered ZIF; OMS: open metal site; SeLiRe: selective ligand removal; CR/SR: complete/surface reconstruction.

A second lesson concerns the fundamental relationship between stability and accessibility. Efficient catalytic turnover often requires direct substrate coordination to catalytically relevant metal centers. In coordinatively saturated frameworks, this process frequently necessitates transient coordination opening, local deligation, or other structural fluctuations that generate temporary adsorption sites. The same coordination bonds that provide structural robustness may also need to undergo reversible perturbation to permit catalytic turnover. Dynamic coordination opening therefore represents not merely a degradation pathway but often an essential elementary step in catalysis. The challenge is balancing macroscopic stability with controlled local lability.

This fundamental tension between accessibility and robustness motivated the development of framework-retaining selective ligand removal (SeLiRe), which embodies a third design principle that catalyst activation should be engineered rather than left to emerge stochastically during operation. By exploiting differences in ligand stability, SeLiRe enables the deliberate generation of open metal sites and hierarchical transport pathways while maintaining structural determinism. More broadly, this approach demonstrates that postsynthetic transformations can provide greater control over catalytic functionality than direct synthesis alone.

A fourth lesson emerges from the dynamic nature of these interfaces under operating conditions. Potential-driven adsorption, hydroxylation, coordination changes, and framework reconstruction can fundamentally alter the active site relative to the as-synthesized material. Consequently, active sites should be defined by their turnover state structure rather than solely by their initial crystallographic configuration. However, identifying these chemically relevant states remains technically daunting. Current *operando* techniques, such as XAFS, Raman, and TRIR, often struggle to capture fast dynamic coordination changes involving subnanosecond transient intermediates. Note that these bulk-averaging techniques suffer from severely low signal-to-noise ratios when attempting to differentiate sparse surface-active species from the overwhelming signals of the bulk MOF matrix.

Taken together, these observations suggest a broader shift in catalyst design philosophy. The objective is not simply to create increasingly complex static structures nor to prevent structural evolution at all costs. Rather, the challenge is to engineer catalysts whose dynamic transformations remain predictable, controllable, and mechanistically interpretable. In this sense, catalyst reconstruction should not be viewed solely as a degradation pathway but as a design parameter that can be deliberately exploited to access new catalytic states.

We therefore propose that the principal value of MOFs lies not in their ability to remain structurally invariant during catalysis but in their unique capacity to bridge structure, dynamics, and mechanism with atomic level precision. Looking forward, engineering predictable dynamic active sites will require overcoming the aforementioned analytical bottlenecks. We anticipate that emerging advanced techniques, such as *in situ* 3D electron diffraction for resolving local defect structures, ambient-pressure/time-resolved X-ray applications for probing delicate solid–liquid interfaces and tracking ultrafast dynamics, will be pivotal in advancing this field. While this perspective has emerged from MOF catalysis, we believe it reflects a broader transition occurring across heterogeneous catalysis, where understanding and controlling catalyst evolution has become as important as controlling its initial crystallographic structure itself.
